# Peroxisome quality control and dysregulated lipid metabolism in neurodegenerative diseases

**DOI:** 10.1038/s12276-020-00503-9

**Published:** 2020-09-11

**Authors:** Doo Sin Jo, Na Yeon Park, Dong-Hyung Cho

**Affiliations:** 1 Brain Science and Engineering Institute, Kyungpook National University, Daegu, 41566 Republic of Korea; 2 School of Life Sciences, Kyungpook National University, Daegu, 41566 Republic of Korea

**Keywords:** Pexophagy, Neurodegenerative diseases

## Abstract

In recent decades, the role of the peroxisome in physiology and disease conditions has become increasingly important. Together with the mitochondria and other cellular organelles, peroxisomes support key metabolic platforms for the oxidation of various fatty acids and regulate redox conditions. In addition, peroxisomes contribute to the biosynthesis of essential lipid molecules, such as bile acid, cholesterol, docosahexaenoic acid, and plasmalogen. Therefore, the quality control mechanisms that regulate peroxisome biogenesis and degradation are important for cellular homeostasis. Current evidence indicates that peroxisomal function is often reduced or dysregulated in various human disease conditions, such as neurodegenerative diseases. Here, we review the recent progress that has been made toward understanding the quality control systems that regulate peroxisomes and their pathological implications.

## Neurodegenerative diseases: The role of peroxisome organelles

Systematic studies of cellular organelles called peroxisomes are needed to determine their influence on the progression of neurodegenerative diseases. Peroxisomes play vital roles in biological processes including the metabolism of lipids and reactive oxygen species, and the synthesis of key molecules, including bile acid and cholesterol. Disruption to peroxisome activity has been linked to metabolic disorders, cancers and neurodegenerative conditions. Dong-Hyung Cho at Kyungpook National University in Daegu, South Korea, and coworkers reviewed current understanding of peroxisome regulation, with a particular focus on brain disorders. The quantity and activity of peroxisomes alter according to environmental and stress cues. The brain is lipid-rich, and even small changes in fatty acid composition may influence neuronal function. Changes in fatty acid metabolism are found in early stage Alzheimer’s and Parkinson’s diseases, but whether peroxisome disruption is responsible requires clarification.

## Introduction

Peroxisomes contain hydrogen peroxide-generating oxidases and were first described by Christian De Duve in the 1960s^1,2^. Peroxisomes are tiny (0.2–1-μm-diameter), single-membrane-bound organelles, and hundreds of peroxisomes can exist in a single mammalian cell^2^. They are highly dynamic and ubiquitous organelles that communicate with other organelles, including mitochondria, the endoplasmic reticulum (ER), lysosome, and lipid droplets, through direct interactions^3^. Peroxisomes function as multipurpose organelles in both catabolic and anabolic pathways, providing different responses in various cells. Peroxisomes play particularly important roles in lipid metabolism, ether-phospholipid biosynthesis, and reactive oxygen species (ROS) metabolism^3^. In cooperation with mitochondria, peroxisomes play important roles in fatty acid oxidation (FAO) and fatty acid production. Moreover, peroxisomes function as signaling platforms for the innate immune response and inflammatory signaling^4,5^. Unsurprisingly, peroxisome dysregulation has been associated with various human disorders, such as metabolic diseases, cancer, and neurodegenerative diseases, as well as peroxisome biogenesis disorders (PBDs)^2^. Zellweger syndrome, one of the three PBDs, is a rare congenital disorder characterized by a reduction in the number of or absence of functional peroxisomes in the cells of an individual. Zellweger syndrome can manifest as a reduction in central nervous system myelin and postdevelopmental sensorineuronal degeneration^6^. To avoid repetition, we refer to our recently published article on PBDs and Zellweger syndrome^7^.

The quality and quantity of peroxisomes are regulated in response to changes in the environment to maintain optimal peroxisome numbers and function^7,8^. Peroxisome regulation consists of active processes that modulate peroxisome abundance, including peroxisome biogenesis and degradation (pexophagy). Peroxisomes can be generated through the growth and division of pre-existing peroxisomes or through de novo synthesis, which requires the fusion of two preperoxisomal vesicles, which are generated by the ER and mitochondria^9^. The growth and division of pre-existing peroxisomes are mediated by elongation factors and fission regulators^10^. These processes are tightly regulated by peroxisome biogenesis factors, known as peroxins (PEXs), and peroxisomal membrane proteins (PMPs)^11^. Selective autophagy of cellular organelles is an important process that maintains homeostasis during various internal and external stress responses. Pexophagy, which refers to the selective autophagic degradation of peroxisomes, can be activated to eliminate dysfunctional or superfluous peroxisomes^3,7^ and is triggered by both stress conditions, such as starvation and hypoxia, and peroxisomal dysfunction, to maintain peroxisome homeostasis^12,13^.

Peroxisomes are essential for cellular redox status and lipid metabolism; however, the physiologic and pathologic roles of peroxisomes remain poorly understood, especially compared with those of mitochondria. Therefore, in this review, we highlight the current understanding regarding the roles played by peroxisome quality control and lipid metabolic dysfunction in neurodegenerative diseases.

## Peroxisome functions

### Lipid metabolism

FAO is tightly regulated at several steps in the oxidation pathway to achieve a balanced energy production and expenditure system. The degradation of oxidized fatty acids occurs in peroxisomes and mitochondria. The β-oxidation of short-, medium-, and long-chain fatty acids predominantly occurs in the mitochondria under physiological conditions. However, the oxidation of specialized fatty acids occurs in peroxisomes, including very-long-chain fatty acids (VLCFAs, i.e., C22:0, C24:0, and C26:0), pristanic acid, and di- or tri-hydroxycholestanoic acids, which cannot be oxidized by mitochondria^14^. During this process, fatty acids undergo successive rounds of FAO, which involves 2-carbon chain-shortening processes. Peroxisomes are involved not only in catabolic processes but also in anabolic processes, including the synthesis of bile acid, docosahexaenoic acid (DHA), cholesterol, and ether phospholipids^14,15^. Ether lipids account for ~20% of all phospholipids in humans, and plasmalogens are particularly abundant in the heart and brain, where they form cell membranes and mediate signals^16,17^. Plasmalogen biosynthesis is initiated in the peroxisome by the enzymes glyceronephosphate *O*-acyltransferase (GNPAT) and alkylglycerone phosphate synthase and is completed in the ER. Fluorescence anisotropy of membrane-bound fluorophores, which indicates increased membrane lipid mobility, has been consistently demonstrated in plasmalogen-deficient cells^16,18^. Remarkably, plasmalogen-deficient cells are more sensitive to ROS and cell death than wild-type cells^19^. Several neurodegenerative disorders have been associated with reduced brain plasmalogen levels. Changes in peroxisomal function in oligodendrocytes may be the primary pathologic factor that results in demyelination, one of the common phenotypes of PBDs, such as Zellweger syndrome^20^. Therefore, the demyelination observed in PBD patients may be due to the depletion of plasmalogen, which is the major component of normal myelin membranes, as a result of VLCFA accumulation in membrane lipids^21^.

Peroxisomes, together with the ER, are also essential for DHA synthesis. The DHA synthesis rate in fibroblasts derived from Zellweger syndrome patients was found to be <5% of that in control fibroblasts^22^. In addition, DHA facilitates peroxisomal division by promoting the oligomerization of peroxisomal biogenesis factor 11 beta (PEX11β), resulting in the initiation of peroxisome elongation^23^. Bile acid intermediates are converted to taurine or glycine conjugates by bile acid-CoA:amino acid N-acyltransferase in peroxisomes^15^. Deficiencies in ATP binding cassette subfamily D member 3 (ABCD3), an ABC transporter found in the peroxisomal membrane, result in bile acid synthesis abnormalities. Analysis of *Abcd3* knockout mice revealed reduced levels of mature C24 bile acid^24^. Cholesterol is an essential determinant of membrane fluidity, permeability, and organization in animal cells. *PEX2* deficiency has been associated with ER stress-induced pathway activation, leading to the dysregulation of the endogenous sterol response mechanism and decreased cholesterol levels in the plasma and liver^25^. In addition, disruption of critical peroxisome genes, such as *PEX1*, results in cholesterol accumulation in the lysosome lumen^26^.

### Redox homeostasis

Redox imbalances are strongly associated with human disease initiation and progression, including neurodegenerative diseases^27,28^. Peroxisomes have emerged as a central source of redox imbalance, affecting ROS generation and scavenging, owing to the similar functions of peroxisomes and mitochondria^29^. Notably, peroxisomes account for ~20% of total cellular oxygen consumption and up to 35% of total H_2_O_2_ generation in mammalian tissues^30^. In addition, peroxisomes are associated with the initiation of the cellular oxidative damage response. Deficiencies in peroxisomal antioxidant proteins, such as catalase, can perturb the mitochondrial redox potential^31^. Furthermore, local oxidative damage to peroxisomes eventually results in mitochondrial dysfunction and cell death^5,28^, implicating that peroxisomes act as upstream initiators of mitochondrial ROS signaling. Peroxisomes also contain several oxidases that can generate various ROS, such as superoxide radicals and hydroxyl radicals^5,14^. Antioxidants are essential for scavenging harmful ROS produced in the peroxisome to maintain the redox balance in cells. In addition to oxidases, peroxisomes also contain other antioxidant enzymes, such as catalase, superoxide dismutase 1 (SOD1), peroxiredoxin 5 (Prx5), S-transferase kappa, epoxide hydrolase, and glutathione peroxidase (GPx)^14,32^. Together, these antioxidant enzymes are responsible for inhibiting excessive ROS generation by peroxisomal oxidases^14,32^. Catalase is a heme-containing enzyme and represents the most abundant peroxisomal antioxidant^14^. Prx5 has a cytoprotective effect against H_2_O_2_- and lipid hydroperoxide-generated oxidative stress^33^. GPx reduces lipid hydroperoxides to their corresponding alcohols and reduces free H_2_O_2_ to form water^14^. Recently, several peroxisomal proteins, such as LonP2, insulin-degrading enzyme, and PEX11β, have been suggested to contribute to the maintenance of peroxisomal redox homeostasis, similar to the abovementioned antioxidants^34–36^.

## Peroxisomal quality control

Peroxisomal quality and quantity are regulated in response to environmental changes to maintain the optimal numbers and functions of peroxisomes^7^. Both peroxisome biogenesis and pexophagy control the number of peroxisomes.

### Peroxisome biogenesis

**Fig. 1:**
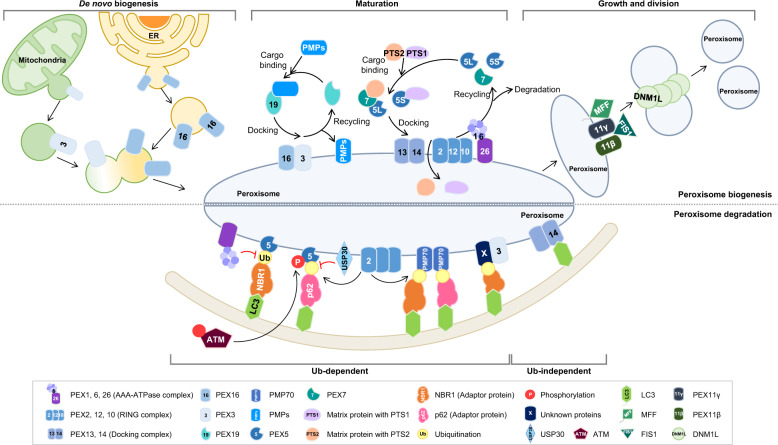
Schematics of the peroxisome biogenesis and degradation systems. The number of peroxisomes is regulated by de novo biogenesis and the growth and division of pre-existing organelles. First, peroxisomes can be formed by the maturation of preperoxisomal vesicles that emerge from the ER or mitochondria and contain peroxisomal membrane proteins, including PEX16, PEX3, and PEX14. Preperoxisomal vesicle fusion results in the generation of mature peroxisomes mediated by PEX19. Second, peroxisomes can be formed by the elongation and division of mature peroxisomes, which are cleaved by the proteins PEX11, Fis1, MFF, and DNM1L (top). Pexophagy is regulated by ubiquitination-dependent and ubiquitination-independent pathways. The ubiquitination of the cytosolic region of peroxisomes triggers their degradation by pexophagy. During oxidative stress, ATM interacts with and phosphorylates PEX5, which promotes PEX5 ubiquitination by PEX2. Ubiquitinated PEX5 is recognized by p62, which recruits the autophagosome. The peroxisomal AAA-type ATPase complex, PEX1, PEX6, and PEX26, prevents pexophagy by regulating the accumulation of ubiquitinated PEX5. During amino acid starvation conditions, PEX2 regulates the ubiquitination of PEX5 and PMP70, increasing pexophagy in an NBR1-dependent manner. USP30 prevents pexophagy by counteracting PEX2. In contrast, PEX14 directly interacts with LC3 under nutrient deprivation conditions (bottom).

The number of peroxisomes can be controlled by the de novo biogenesis of peroxisomes through the fusion of mitochondria- and ER-derived preperoxisomal vesicles and the growth and division of pre-existing organelles (Fig. 1). These processes are complicated and are tightly regulated by more than 30 PEX proteins^37,38^. PEX proteins play important roles in many biological processes, such as targeting PMPs to peroxisomes, controlling peroxisomal size, and maintaining peroxisomal functions^37–39^.

Preperoxisomal vesicles emerge from a distinct subdomain of the ER that is enriched in PEX16^11^. Peroxisome biogenesis is regulated by the insertion of PEX16 into the ER membrane and the recruitment of another peroxin, PEX3, which serves as a docking factor for PEX19 on peroxisomes. PEX3 binds newly synthesized PMPs in the cytosol and delivers them to the peroxisome for insertion into the membrane^40,41^. Recently, PEX3 and PEX14 were reported to be selectively released into vesicular preperoxisomal structures. Mitochondria-derived vesicles containing PEX3 and PEX14 fuse with ER-derived vesicles; thus, newly generated peroxisomes can obtain membranes from diverse sources and expand their functional linkages to the mitochondria and the ER^9^.

Alternatively, peroxisomes can be formed through elongation and division processes. Mature peroxisomes are elongated and segregated by the cooperation between the PEX11 family and fission mitochondrial 1 (Fis1) and mitochondrial fission factor (MFF), which recruit the GTPase dynamin1-like (DNM1L) to cleave the peroxisomal membrane^42^. The peroxisome maturation process is facilitated by the import of matrix proteins by receptor proteins and peroxisome-targeting signals^43^. Peroxisomal matrix proteins are synthesized in the cytosol and transported to the peroxisome matrix. Two types of targeting signals direct most proteins to the peroxisome matrix. Most peroxisomal matrix proteins contain a C-terminal peroxisomal targeting signal (PTS1), although some contain the less common N-terminal PTS2^44,45^. These signals are recognized by the soluble import receptors PEX5 and PEX7^46,47^. PEX5 binds to the C-terminus of PTS1 and imports the target protein into the peroxisome^48^. PEX14 and PEX13 comprise the protein import machinery that forms the docking complex that binds PEX5-PTS1-containing proteins^48^. During the last step of the matrix protein import cycle, PEX5 is recycled back into the cytoplasm in a monoubiquitin-dependent manner for further rounds of import or is subjected to proteasome-mediated turnover of dysfunctional receptors^49^.

Peroxisomal proteins are influenced by several gene regulators at the transcriptional level, including peroxisome proliferator-activated receptors (PPARs), which represent the best-characterized nuclear receptors and are essential for regulating the transcriptional activation of peroxisomal proteins, especially peroxisomal beta-oxidation^50^. PPARs have been reported to act as autophagy regulators, even under feeding conditions^51^.

### Peroxisomal degradation (pexophagy)

Autophagy is responsible for degrading cellular components and initiated by the nucleation of isolated membranes, which gradually elongate to form a mature autophagosome that captures cytosolic materials. Upon maturation, the outer membrane of the autophagosome fuses with the lysosome membrane, leading to the degradation of autophagosomal contents. Autophagy has long been considered a nonselective degradation process; however, recent research has indicated that autophagy selectively eliminates specific components, referred to as selective autophagy, including peroxisomes (pexophagy), mitochondria (mitophagy), lysosomes (lysophagy), and ER (reticulophagy)^52^. Autophagy is a sequential process that is primarily regulated by autophagy-related genes (ATGs) and various adaptor/receptor proteins, including sequestosome 1 (SQSTM1/p62), optineurin (OPTN), NBR1, NDP52, NIX, and FUNDC1^53^.

Peroxisomal quality control is regulated by two distinct mechanisms. During peroxisomal degradation, 70–80% of excess peroxisomes are degraded through autophagy, whereas the remaining 20–30% of peroxisomes are degraded by other processes mediated by peroxisomal LonP2 and 15-lipoxygenase-1^54,55^. Recent advances in the understanding of selective autophagy have suggested that the ubiquitination of membrane proteins found in specific organelles mediates the initiation of selective autophagy^56–58^. According to this notion, the ubiquitination of the cytosolic region of peroxisome-associated proteins triggers peroxisome degradation by pexophagy (Fig. 1, Table 1). Kim et al. showed that the ectopic expression of PEX3 or PMP34 fused to ubiquitin on the cytosolic face decreases the number of peroxisomes and that the ubiquitin signal on the peroxisomal membrane is recognized by p62 or NBR1, which targets peroxisomes to the autophagosome^59^. Moreover, the exogenous expression of NBR1 induces peroxisome clustering and targeting to lysosomes, promoting pexophagy^60^. NBR1 has a similar domain composition as p62, consisting of a PB1 domain at the N-terminus, a ZZ domain in the coiled coil, an LIR motif in the middle part of the molecule, an amphipathic alpha-helical J domain (JUBA) and a UBA domain at the C-terminus^61,62^. The PB1 domain of NBR1 mediates interactions with p62, and both the JUVA and UBA domains are involved in the localization of NBR1 on peroxisomes. Mutation studies have shown that the JUBA, UBA, and LIR domains of NBR1 contribute to pexophagy^60^. Among the PEX proteins, PEX5 closely regulates pexophagy. The inhibition of PEX5 recruitment by PEX14 depletion significantly reduces pexophagy^60^. During the PMP import cycle, PEX5 is regulated in a ubiquitination-dependent manner, whereas polyubiquitinated PEX5 is degraded by the proteasome system. Nordgren et al. showed that export-deficient monoubiquitinated PEX5, which maintains monoubiquitinated PEX5 at the membrane long enough to be recognized by the autophagic machinery, promotes peroxisomal removal^63^. In addition, Zhang et al. also reported that PEX5 binds to the protein ataxia-telangiectasia mutated (ATM). During oxidative stress, ATM directly phosphorylates PEX5 at Ser 141, which subsequently promotes PEX5 monoubiquitination at Lys 209. Ubiquitinated PEX5 is then recognized by p62, which recruits the autophagosome^64^. The peroxisomal AAA–ATPase complex consisting of PEX1, PEX6, and PEX26 prevents pexophagy and peroxisome biogenesis disorder development^65^. The loss of the ATPase associated with diverse cellular activities (AAA)–ATPase complex, which is required to cycle PEX5 for PMP import, results in the accumulation of ubiquitinated PEX5 on the peroxisomal membrane, triggering pexophagy^65^. Each of the three RING peroxins, namely, PEX2, PEX10, and PEX12, exhibits ubiquitin-protein isopeptide ligase activity. Members of the E2D (UbcH5) family act as specialized ubiquitin-conjugating enzymes that mediate the ubiquitination of PEX5^66^. PEX5 and PMP70 are ubiquitinated by PEX2 during pexophagy triggered by amino acid starvation. PEX2 expression results in the gross ubiquitination of peroxisomes and pexophagy in an NBR1-dependent manner^67^. Conversely, the deubiquitinating enzyme USP30 prevents pexophagy by counteracting the activity of PEX2. USP30, which is known as a mitophagy regulator, can also be localized to peroxisomes^68,69^. USP30 overexpression prevents pexophagy during amino acid starvation by counteracting the PEX2-mediated ubiquitination of PEX5 and PMP70, whereas USP30 depletion results in pexophagy induction, even under basal conditions^70^. PEX14 and PEX13 comprise the protein import machinery that serves as a docking complex for PEX5. PEX14 has been suggested to directly interact with the LC3II autophagosomal protein^71^. During this process, PEX14 preferentially interacts with LC3 rather than PEX5 under nutrient-deprived conditions^71^. In addition to PEX14, PEX3 may target peroxisomes for pexophagy. In PEX3-overexpressing cells, peroxisomes are ubiquitinated and degraded via an NBR1-dependent process^58^. Taken together, these previous studies describe the regulatory mechanisms associated with ubiquitination-dependent pexophagy. However, the precise regulatory mechanisms that control the ubiquitination process require further investigation.

**Table 1:** Peroxisomal proteins involved in peroxisome quality control.

	Gene	Function	Reference
*Peroxisome biogenesis*			
De novo biogenesis			
PEX3	Peroxisomal Biogenesis Factor 3	Formation of preperoxisomal vesicles	^8,39,40^
PEX16	Peroxisomal Biogenesis Factor 16	Formation of preperoxisomal vesicles	^10,39,40^
PEX19	Peroxisomal Biogenesis Factor 19	Receptor for mPTS membrane protein	^39,40^
Maturation			
PEX5	Peroxisomal Biogenesis Factor 5	Receptor for PTS1 matrix protein	^45,47,48^
PEX7	Peroxisomal Biogenesis Factor 7	Receptor for PTS2 matrix protein	^46^
PEX1	Peroxisomal Biogenesis Factor 1	AAA–ATPase complex for PEX5 recycling	^48^
PEX6	Peroxisomal Biogenesis Factor 6		^48^
PEX26	Peroxisomal Biogenesis Factor 26		^48^
PEX2	Peroxisomal Biogenesis Factor 2	RING complex for PEX5 ubiquitination	^48,65^
PEX10	Peroxisomal Biogenesis Factor 10		^48,65^
PEX12	Peroxisomal Biogenesis Factor 12		^48,65^
PEX13	Peroxisomal Biogenesis Factor 13	Docking complex for matrix protein import	^8,47^
PEX14	Peroxisomal Biogenesis Factor 14		^22,41^
PEX3	Peroxisomal Biogenesis Factor 3	Docking factor for PEX19	^8,39,40^
PEX16	Peroxisomal Biogenesis Factor 16	Recruitment of PEX3	^10,39,40^
Growth and division			
PEX11β	Peroxisomal Biogenesis Factor 11 Beta	Interaction with DNM1L	^9^
PEX11γ	Peroxisomal Biogenesis Factor 11 Gamma	Elongation of peroxisome and attraction of FIS1 and MFF	^41^
FIS1	Fission, Mitochondrial 1	Interaction with PEX11γ and recruitment of DNM1L	^41^
MFF	Mitochondrial Fission Factor	Interaction with PEX11γ and recruitment of DNM1L	^41^
DNM1L	Dynamin 1 Like	Cleavage of peroxisomal membrane	^9,41^
*Peroxisome degradation*			
Ub-dependent			
PEX5	Peroxisomal Biogenesis Factor 5	Target of phosphorylation and ubiquitination	^59,62–64,66,69,70^
PMP70	ATP Binding Cassette Subfamily D Member 3	Target of ubiquitination	^66,69^
ATM	ATM Serine/Threonine Kinase	Phosphorylation of PEX5	^63^
PEX1	Peroxisomal Biogenesis Factor 1	Recycling of PEX5	^64^
PEX26	Peroxisomal Biogenesis Factor 26	Recycling of PEX5	^64^
PEX2	Peroxisomal Biogenesis Factor 2	Ubiquitination of PEX5 and PMP70	^65,66,69^
USP30	Ubiquitin Specific Peptidase 30	Removal of ubiquitin from PEX5 and PMP70	^68,69^
p62/SQSTM1	Sequestosome 1	Ubiquitin-binding protein	^52,58,63^
NBR1	NBR1 Autophagy Cargo Receptor	Ubiquitin-binding protein	^57–61,66^
Ub-independent			
PEX3	Peroxisomal Biogenesis Factor 3	Increase of peroxisomal ubiquitination	^57^
PEX14	Peroxisomal Biogenesis Factor 14	Interaction with LC3II	^57,59,70^

Several methods have using various model systems been proposed to study pexophagy. Pexophagy is a dynamic process that ends in the lysosome, which has the most acidic cellular microenvironment of any organelle. This property of lysosomes has led to the development of peroxisome-targeted forms of pH-dependent systems to monitor pexophagy. To observe and quantify pexophagy activity, Nazrko and coworkers utilized an mRFP-EGFP protein containing the PTS1 domain^72^. Similarly, Deosaran et al. used a tandem chimera of mCherry and EGFP fused to the peroxisome-membrane-targeting sequence of PEX26^60^. The GFP fluorescence of the fused protein is quickly quenched, whereas the mRFP fluorescence exhibits more stability under acidic conditions in the lysosome^73^. In addition to these tandem systems, we also developed a pexophagy assay model using a pH-sensitive pexo-dKeima generated by fusing the PTS1 sequence to the dKeima protein^74^. The dKeima protein is a pH-sensitive, dual-excitation, ratiometric fluorescent protein that exhibits lysosomal protease resistance. At the physiological pH of the peroxisome (pH 6.9–7.1), shorter-wavelength excitation predominates. At the end of pexophagy, pexo-Keima undergoes a gradual shift to longer-wavelength excitation within the acidic lysosomal environment (pH 4.5)^74–76^.

## Peroxisome dysfunction in neurodegenerative diseases

The brain is a lipid-rich organ, with membrane lipids constituting 50–60% of the total solid brain matter^77^. Therefore, slight alterations in fatty acid composition may lead to considerable changes in neuronal function. Several inherited peroxisomal disorders have been associated with severe neurologic dysfunctions, including hypotonia, seizures, cerebellar ataxia, sensory impairment, and developmental deficits^78^. Recent studies have suggested that peroxisomal metabolic function is also disrupted in age-related neurological disorders, including Alzheimer’s disease (AD) and Parkinson’s disease (PD)^28,32^. Therefore, in this review, we focus on metabolic dysregulation associated with peroxisome dysfunction in AD and PD.

### Peroxisome dysfunction in Alzheimer’s disease

AD is the most common neurological disorder that affects the elderly population and is clinically characterized by the progressive deterioration of cognition, behavior and functionality, leading to significant impairment of activities of daily living^79^. Primary histopathologic lesions associated with AD pathology indicate neuroinflammation and neuronal loss, which are accompanied by beta-amyloid (Aβ) plaques and neurofibrillary tangles^80–82^. The toxic properties of Aβ plaques are mediated by diverse mechanisms, including oxidative stress, inflammation, synaptic dysfunction, and excitotoxicity^83^. Tauopathy is another widely accepted component of AD pathology. When tau protein becomes highly phosphorylated, it aggregates, inhibiting microtubule function, impairing neuronal axonal transport, and thus leading to neuronal cytotoxicity^84^. Emerging evidence has suggested that in addition to Aβ and tau, inflammation may play a causal role in AD pathogenesis^80^. Serial studies of lipid metabolism have shown that lipid alterations can be detected during early AD progression^85–87^. Remarkably, a significant and selective decrease in plasmalogen can be observed in postmortem brain samples from AD patients^88,89^. Kuo et al. measured the levels of VLCFAs in cortical brain regions affected by AD and found that VLCFAs, such as C24:0 and C26:0, accumulate in all cortical areas except the parasubiculum^87^. In addition, increased VLCFA levels have been associated with the presence of neurofibrillary tangles^87,89^. Consistent with this finding, total plasmalogen concentrations have been found to be significantly decreased in the gyrus frontalis region of AD patients^87,88^.

Notably, cells from patients with Zellweger syndrome, a PBD, show lysosomal cholesterol accumulation^26^. Several epidemiologic studies have indicated that hypercholesterolemia is closely associated with AD pathology, although the exact mechanism through which cholesterol affects AD pathogenesis is largely unknown^90^. Plasma cholesterol levels are ~10% higher in AD patients than in normal controls, and several genes associated with hypercholesterolemia, such as *ApoE4*, increase the incidence of AD^91,92^. ApoE4, a strong genetic risk factor for late, sporadic AD onset, transports cholesterol, and other lipid components into neurons^93^. Cholesterol and oxysterol imbalances can cause alterations in cell membrane properties and increase intracellular cholesterol levels, enhancing the activities of beta-secretase 1 (BACE1) and increasing γ-secretase levels, which are associated with increased levels of Aβ generation^94^.

Similar to plasmalogen and cholesterol, DHA has also been identified as a causal factor in AD pathogenesis and progression^95^. DHA plays an important role in normal neurological development, especially in the brain and retina^96^. However, the DHA concentration has been shown to be reduced in the hippocampus in AD^85^, and the levels of DHA in the hippocampus, frontal cortex and temporal cortex are lower in AD patients than healthy individuals^84,97^. DHA inhibits AD pathogenesis by attenuating the Aβ burden, inhibiting tau phosphorylation and decreasing neuroinflammation^98,99^. Accordingly, various studies have suggested that DHA supplementation can effectively reduce key AD-associated risk factors. A DHA-enriched diet can increase cerebral blood volume and decrease vascular Aβ deposition, leading to selective changes in the phospholipid profiles of different brain regions in mouse models of AD^100,101^. In addition, DHA can suppress proinflammatory cytokine expression in neurons^97,102^. Neuroprotectin D1, a bioactive metabolite of DHA, inhibits neuroinflammation and toxicity^103^.

PPARs, which act as lipid sensors and peroxisomal gene activators, are associated with the transcriptional control of genes that regulate metabolism^104^. PPAR agonists, such as pioglitazone, can ameliorate AD-related pathology and improve cognition by decreasing Aβ production^105,106^. PPAR agonists also inhibit inflammatory gene expression and immune responses and inhibit the secretion of proinflammatory cytokines^107,108^. Icariin, a prenylated flavonol glycoside found in various medicinal herbs, attenuates M1 microglial activation and Aβ plaque formation in the hippocampus and prefrontal cortex by increasing PPARγ levels in an AD mouse model^109,110^. Neuroinflammation also plays a role in AD pathophysiology and is considered a promising target for AD treatment^111^.

### Peroxisome dysfunction in Parkinson’s disease

PD is the second most common neurodegenerative disease after AD and is characterized by the selective loss of dopaminergic neurons in the substantia nigra and the underproduction of dopamine coupled with α-synuclein (SNCA) accumulation. In recent decades, studies have confirmed that various genetic factors, including *DJ‐1, LRRK2*, *Parkin, PINK 1, SNCA*, and *VPS35*, contribute to the complex pathogenesis of PD^112–114^.

Postmortem lipid composition analysis of lipid rafts from the frontal cortices of PD patients have indicated remarkable reductions in polyunsaturated fatty acid contents, including DHA and arachidonic acid, whereas saturated fatty acid levels are enhanced in the brains of PD patients compared with the brains of control subjects^115^. Another study that examined serum lipid profiles in PD patients showed lower levels of total cholesterol and triglycerides in PD patients than in control individuals^116^. Furthermore, several studies have indicated a relationship among the levels of peroxisomal lipids, such as cholesterol, the use of drugs that regulate cholesterol levels and PD development^117,118^. Ethanolamine plasmalogens are also diminished in the blood and brains of PD patients, and supplementation with the ethanolamine plasmalogen precursor PPI-1011 helps reverse striatal dopamine loss in a PD mouse model^119,120^. Thus, these lipids may be used as markers of PD severity. The neuroprotective effects of PPAR agonists have been assessed in several PD models as in AD models^121^. Pioglitazone and rosiglitazone, which were originally designed as PPARγ agonists, have been shown to block dopaminergic neurodegeneration and reduce astrocytic and microglial activation^122^. In addition, PPAR α/γ agonists, such as fenofibrate and MHY908, prevent neurotoxicity in a mouse model of 1-methyl-4-phenyl-1,2,3,6-tetrahydropyridine (MPTP)-induced PD^123,124^. The role played by oxidative stress in dopaminergic neuron degeneration has been extensively studied. Oxidative damage to lipids, proteins, and DNA occurs during PD, and the toxic products generated by oxidative damage can react with proteins, proteasome systems, and autophagy, impairing cell viability^125–128^.

Increased ROS production combined with defects in peroxisomal antioxidant mechanisms and the accumulation of lipid intermediates in the peroxisomal FAO system has been suggested to alter mitochondrial function and may contribute to PD pathogenesis. Marked mitochondrial abnormalities have been observed in several organs in *PEX5-*deficient mice^129^. In addition, deficiencies in peroxisome biogenesis associated with a mutation in PEX3 prevents the binding of SNCA to lipid droplets in lipid-loaded yeast^130^. Recently, our group also showed that HSPA9/mortalin depletion induces pexophagy by increasing peroxisomal ROS^74^. The overexpression of wild-type HSPA9 reverses peroxisome loss, whereas an HSPA9 mutant associated with PD fails to rescue HSPA9-depleted neuronal cells^74^.

Although peroxisome abundance and lipid metabolism play roles in several pathological neuronal conditions, it remains unclear whether these conditions represent secondary changes associated with general cellular dysfunction. Therefore, to better understand the roles played by peroxisomes in neurodegenerative diseases, further studies are warranted.

## Conclusion and perspective

Peroxisomes are key metabolic organelles that have protective functions and wide-reaching impacts on human health and may contribute to a large number of globally important human diseases. Further systematic studies are necessary to determine whether peroxisome alterations/dysfunctions contribute to disease etiology. In addition, the functional correlations between disease pathogenesis and alterations in peroxisome physiology remain to be elucidated. Emerging research areas include the roles played by peroxisomes in cellular redox balance, metabolic balance, and pexophagy. Because peroxisomes are one of the most unexplored subcellular organelles in eukaryotic cells, the continued exploration of their functional significance is likely to reveal additional and useful information in the future.
